# Nanogels as a Versatile Drug Delivery System for Brain Cancer

**DOI:** 10.3390/gels7020063

**Published:** 2021-05-26

**Authors:** Brielle Stawicki, Tyler Schacher, Hyunah Cho

**Affiliations:** School of Pharmacy and Health Sciences, Fairleigh Dickinson University, 230 Park Ave., Florham Park, NJ 07932, USA; brielled@student.fdu.edu (B.S.); tschach@student.fdu.edu (T.S.)

**Keywords:** nanogel, drug delivery, brain cancer

## Abstract

Chemotherapy and radiation remain as mainstays in the treatment of a variety of cancers globally, yet some therapies exhibit limited specificity and result in harsh side effects in patients. Brain tissue differs from other tissue due to restrictions from the blood–brain barrier, thus systemic treatment options are limited. The focus of this review is on nanogels as local and systemic drug delivery systems in the treatment of brain cancer. Nanogels are a unique local or systemic drug delivery system that is tailorable and consists of a three-dimensional polymeric network formed via physical or chemical assembly. For example, thermosensitive nanogels show promise in their ability to incorporate therapeutic agents in nano-structured matrices, be applied in the forms of sprays or sols to the area from which a tumor has been removed, form adhesive gels to fill the cavity and deliver treatment locally. Their usage does come with complications, such as handling, storage, chemical stability, and degradation. Despite these limitations, the current ongoing development of nanogels allows patient-centered treatment that can be considered as a promising tool for the management of brain cancer.

## 1. Introduction

With the continuous input of scientific medical research, cancer treatments have improved substantially over the past decade. Brain cancer proposes a unique situation, having similarities to other forms of cancer in the body, yet major differences in the diversity of intracranial neoplasms, genetic heterogeneity, complexity of the organ in which it resides and physiological features of the cranial cavity that limit treatment options [1]. A multitude of brain tumor types exist that are categorized based on their location of origin and malignancy properties, and surgical removal and chemotherapy serve as vital options for the majority of types that are deemed treatable. In 2020, the Central Brain Tumor Registry of the United States reported an overall primary malignant tumor incidence rate of 7.08 per 100,000, an estimated 123,484 cases, and a non-malignant tumor incidence rate of 16.71 per 100,000, 291,927 cases [2,3,4,5]. In 2021, it is projected that approximately 84,170 new cases will be diagnosed in the United States, highlighting the necessity of ever-expanding efficacious treatment options for patients. Brain tumors can be classified based on their location within the cranial cavity, presumptive origin, and microscopic similarities [6]. Internal malignant tumors include the common tumor type, gliomas, which presumptively derive from glial tissues [7]. They are further categorized into astrocytomas and oligodendrogliomas and subdivided into grades based on tumor pathological characteristics. These grades dictate treatment options and responsiveness. Other internal malignant tumors include ependymomas, affecting the ependymal cells of the four ventricles of the brain and the spinal cord canal, and gangliogliomas. Extrinsic malignant tumors, such as meningiomas and schwannomas arise from dura matter and Schwann cells, respectively.

Patients may experience both general and localized symptoms prior to diagnosis and radiographic visualization of their brain tumors. These symptoms include headaches, nausea, seizures, and vomiting due to increased intracranial pressure [7]. The specific symptoms and signs produced by brain tumors vary with the location of the tumor. For example, patients with the tumors located in or subjacent to cortical regions may present with language dysfunction, visual field abnormalities, or focal seizures [8]. Tumors arising in the brain stem may cause rapidly progressing cranial neuropathies as well as motor and sensory deficits [8]. Despite the location of cranial infliction, surgical debulking of the tumor remains a competent first-line treatment option and is used in conjunction with radiotherapy and/or systemic chemotherapy.

A vital consideration that must be taken when beginning systemic chemotherapy for brain malignancies is the blood–brain barrier (BBB) [9]. This barrier consists of a microvascular system that supplies nutrients to the central nervous system. The blood vessels possess unique properties that allow them to vigorously regulate molecules, ions, and cells moving from the blood to the CNS tissues, resulting in CNS homeostasis and the prevention of entrance of toxins and pathogens [9]. Physically, it is composed of continuous capillaries with endothelial cells attached via tight junctions that are able to restrict paracellular diffusion of solutes. P-glycoprotein efflux transporters limit lipophilic solute entrance to the brain. These gatekeeping properties thus also prevent pharmacological substances, such as chemotherapy, from entering and working in brain tissues. The BBB is seen as an obstacle that must be overcome to treat brain metastasis; thus, the developed therapeutic agents are specifically engineered with this in consideration. Generally, therapeutic agents with a molecular mass of <400–600 Da that are lipid soluble have greater BBB penetration [10]. Although a variety of chemotherapy agents are available, not all have the ability to cross the blood–brain barrier, thus limiting treatment options for patients presenting with malignant gliomas. Temozolomide, an alkylating agent, is a viable option. Its mechanism of action consists of the transfer of its alkyl group at the O6and N7 guanine positions causing DNA double strand breaks and apoptosis inside the nucleus of cancer cells [11], Historically, the nitrosoureas (e.g., carmustine, lomustine) and vincristine have been the most widely used class of chemotherapy agents due to their physicochemical properties that enable them to penetrate the BBB and exert therapeutic effects [12,13,14]. Nitrosoureas undergo biotransformation via non-enzymatic decomposition to active metabolites with a mechanism of action similar to alkylating agents. However, these agents are limited by serious nausea, vomiting, and an increased risk of secondary malignancies, due to their overall carcinogenic nature, myelosuppression, infertility, and mucositis [15]. 

Radiation oncology is based on the principles of x-ray machines and directs harmful ionizing radiation to kill cancer cells [16]. Ionizing radiation deposits energy in cancer cells which can directly cause death or result in detrimental genetic mutations. These genetic mutations alter DNA, causing both single and double strand breaks, preventing further tumor growth. As a result of genomic instability, the cells die via apoptosis, necrosis, mitotic catastrophe, autophagy, and other mechanisms [16]. When used in conjunction with neurosurgery, radiation can be used prior to shrink the tumor size, or after to remove the cancerous cells that remain in the area [16]. However, it does not come without its limitations. Radiation commonly results in acute radiation central nervous system toxicity, characterized by nausea, drowsiness, and ataxia. Late effects can be seen 9 months to 10 years after therapy and include focal necrosis, CNS neurological dysfunction, MRI visible white matter alterations, and necrotizing encephalopathy [17]. Cranial radiation may affect other body systems, causing endocrine abnormalities due to a disruption of normal pituitary/hypothalamic axis function and leading to a need for increasing monitoring of anterior and posterior pituitary hormone levels [18].

## 2. Gliadel Wafers for Postsurgical Brain Cancer Treatment

The realization of less-than-ideal characteristics of available radiation and systemic chemotherapy treatment options has led to the search for novel adjunctive “implanted” therapies. BCNU (Gliadel) wafer therapy, approved for use in 1996 by the FDA’s Oncology Drug Advisory Committee, was the first implantable drug delivery system used to deliver carmustine, directly to the site of a surgically resected tumor [19]. Upon tumor removal, the wafers are implanted, providing direct treatment and limiting systemic side effects. The wafer is dime-sized and consists of polifeprosan 20, a biodegradable polyanhydride copolymer. Through slow erosion of the polymer matrix, polifeprosan 20 releases carmustine gradually for an extended period of time, approximately 2–3 weeks [20]. Generally, up to 8 wafers are placed in the tumor cavity, each with 7.7 mg of carmustine, for a total dose of 61.6 mg [21]. Westphal et al. demonstrated that the median survival time after gliadel wafer implantation was 13.8 months compared to 11.6 months in the placebo-group patients [22]. A decrease in mortality by 30% in those treated with the gliadel wafers was also reported [22]. Further studies compared the efficacy of the wafer versus classical chemotherapy agents, such as temozolomide, and showed a difference in peak survival. The absolute percentage gain of survival over placebo with gliadel wafers showed peaks at 12 months versus 18 months with temozolomide [23]. Thus, the sequential use of the two agents was proposed and decreed the “Stupp protocol” [24]. Clinical trials have shown an increased median survival time, including Stupp et al., which compared a combination gliadel wafer implantation and temozolomide to temozolomide treatment alone [24]. In a 5 year follow-up, 97% of patients treated with solely temozolomide died, compared to 89% of patients who received combination therapy [24]. The overall survival was 14.6 months in the wafer plus temozolomide group, and 12.1 months for wafer alone [24]. Post-implantation of gliadel wafers, patients should be monitored for adverse effects and complications, including hemotoxicity [25]. There has been a concern associated with an increased risk of intracranial infections, brain abscess, and cerebrospinal fluid leaks. Other side effects include headaches, cerebral oedema, drowsiness, and seizures [26]. When following the Stupp protocol, multiple studies have demonstrated that there were no unexpected adverse effects or increased incidence reported [27,28].

The gliadel wafer is a rigid, disc-shaped wafer compressed from the mixture of spray-dried polyanhydride and carmustine [19]. Though proven modestly effective, gliadel wafer therapy has well-recognized drawbacks, including limited drug loading capability for a single hydrophilic drug, uneven drug release due to the rigid structure incapable of intimate contacting with surrounding tissues and cumbersome to maximally cover the resection cavity (requires cutting/overlapping the wafers). 

Nanogel-based local delivery of chemotherapy has shown great promise in overcoming the weaknesses of gliadel wafer therapy. A thermosensitive nanogel formulation, OncoGel, is a triblock copolymer comprised of poly(D,L-lactide-co-glycolide), (PLGA) and poly(ethylene glycol) (PEG) with the basic structure of PLGA-b-PEG-b-PLGA. OncoGel contains paclitaxel at 6 mg/mL and makes a sol-to-gel transition at 37 °C. OngoGel entered a phase II clinic trial for treating esophageal cancer [29]. Although found to have low toxicity and reduce tumor burden, OncoGel failed in this clinical study due to insufficient efficacy in esophageal cancer. Tyler et al. proved that OncoGel containing 6.3 mg/mL paclitaxel was safe for intracranial injection in 18 Fischer-344 rats bearing glioma and most effective when administered in combination with radiation therapy [30]. Torres et al. used computational mass transport simulations to investigate the effectiveness of paclitaxel delivery from OncoGel [31]. The effective therapeutic concentrations were maintained for > 30 days using OngoGel whereas those were maintained for ca. 4 days for carmustine released from gliadel wafers. This result was simulated due to the controlled release of paclitaxel within the degradation lifetime of the OncoGel matrix. Nanogels are bioadhesive, thus does not require additives to secure it against the cavity surface after brain surgery. Unlike gliadel wafers that require cutting and overlapping wafers to properly cover cavities of different sizes and shapes, the dose of the drugs loaded in nanogels can be easily controlled and adjusted using a syringe to offer patient-centered treatment (considering the size/shape of the tumor resection cavity). In this short review paper, we summarized the desirable properties of nanogels and possible obstacles with their development and use, and highlighted the application of biocompatible nanogels as a drug delivery system in brain cancer.

## 3. Nanogel

### 3.1. Overview and Preparation of Nanogels

**Figure 1:**
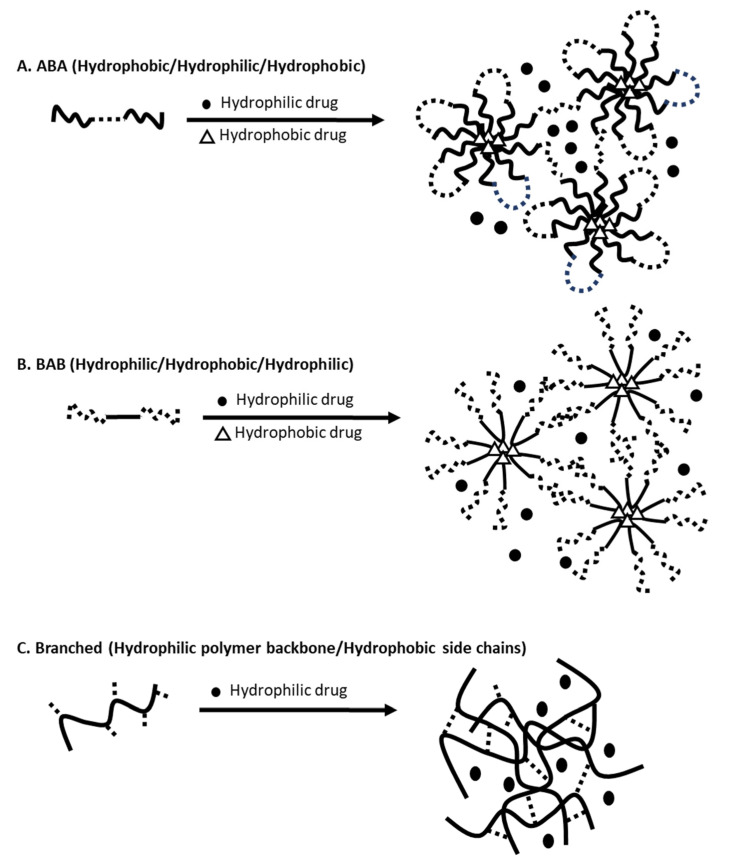
Illustrations of physically self-assembled nanogels loaded with hydrophilic (solid circle) and hydrophobic (empty triangle) drugs using (**A**) ABA, (**B**) BAB, and (**C**) branched polymers (modified from [50]).

Nanogels have a three-dimensional “nanoscopic” structure composed of a variety of natural polymers, synthetic polymers or a combination of both. Nanogels are formed via physical or chemical assemblies of polymers that carry amphiphilic macromolecular chains (Table 1) [32]. Physical assembly relies on the physical interactions/entanglements via hydrogen bonds, electrostatic, van der Waals and hydrophobic interactions, and chemical cross-linking utilizes covalent bonds [32]. Aforementioned nanogels carrying paclitaxel, OncoGel, is one of the examples formed via physical self-assembly [31]. In water, below the critical gelation temperature (CGT), PLGA-b-PEG-b-PLGA (ABA type) copolymers create loops sharing a hydrophobic PLGA center and form nanoscopic micelles (Figure 1A) [33]. Paclitaxel (logP 3.0) is physically entrapped in the core of the micelles. Gelation of PLGA-b-PEG-b-PLGA occurs above the CGT. As the temperature increases, hydrophobic interactions among PLGA segments become stronger, leading to aggregation of micelles, decrease in the mobility of water, and increase in the viscosity (gelation occurs). Akiyoshi et al. physically-assembled nanogels using hydrophobized cholesterol-bearing pullulan that deliver insulin [34]. Nanogels were c.a. 20–30 nm in diameter and self-assembled into nanogels with insulin (Figure 1C) [34]. Another way of physically assembling nanogels is to suspend/immobilize nanoparticles in a hydrogel matrix. Giovannini et al. formed silica nanoparticle (c.a. 100 nm in diameter) and gold nanoparticles (c.a. 80 nm in diameter) and suspended them in Fmoc-galactosamine-based hydrogels [35]. Nanogels carrying silica and gold nanoparticles decreased the premature drug release of loaded drugs as the hydrogels restricted the movement of the nanoparticles and retarded the aggregation of nanoparticles. Overall, the introduction of hydrogels improved the stability of nanoparticles. 

Nanogels can be formed from polymer precursors vial chemical cross-linking that utilizes some functional groups such as disulfide, amine and imine or via photo-induced cross-linking (Table 1) [32]. Ryu et al. developed nanogels composed of polymers that carry an oligoethyleneglycol unit for hydrophilicity and a pyridyl disulfide (PDS)-derived thioethylmethacrylate for the cross-linkability [36]. Disulfide bonds impart structural stability for hydrophobic payloads. Disulfide can reversibly reduce to thiol, as a function of thiol concentrations of the environment. As thiols are highly reduced in cells, by using thiol-disulfide exchange, disulfide bonds are rapidly degraded, releasing the payloads selectively under the reduced condition in cells. Elkassih et al. developed fully degradable disulfide cross-linked nanogels using oxidative radical polymerization of 2,2′-(ethylenedioxy)diethanethiol (EDDET) as a monomer with different cross-linkers, including pentaerythritol tetramercaptoacetate (PETMA) [37]. As the poly(EDDET) backbone repeated structures and cross-linking junctions were composed entirely of disulfide bonds, nanogels were able to degrade into thiols intracellularly in response to the reducing agent glutathione present inside of cells. Amine groups are widely used in the preparation of nanogels because of their high reactivity with carboxylic acids, activated esters, isocyanates and iodides [32]. This technique provides an opportunity to introduce various stimuli-response properties into nanogels by incorporating a diamine cross-linker. Kockelmann et al. developed nanogels carrying imidazoquinolinen using active-ester-containing amphiphilic poly(methacrylate) block copolymers [38]. The amphiphilic reactive ester block copolymers self-assembled into precursor micelles, whose cores were functionalized by mono-amine-bearing entities, cross-linked with pH-degradable bisamines, and finally converted into fully hydrophilic nanogels. The authors stated that these nanogels provided safe and controllable drug delivery strategies in immunotherapy for cancers, considering their slightly acidic environment. Liao et al. developed functionalized polymeric nanogels with pH-responsive benzoic-imine cross-linkages [39]. The polymer was synthesized by one-step cross-linking of the branched poly(ethylenimine)-g-methoxy poly(ethylene glycol) (PEI-g-mPEG) copolymer with hydrophobic terephthalaldehyde (TPA) molecules. The functionalized polymeric nanogels were comprised of multiple hydrophobic benzoic-imine-rich spherical domains covered by positively-charged PEI networks. The external PEI network and mPEG attracted large amounts of water whereas the TPA created the colloidal core more hydrophobic and compact. The nanogels exhibited acid-triggered drug release (indocyanine green incorporated in hydrophobic core via pi-pi stacking) by the cleavage of benzoic-imine bonds in response to pH reduction from 7.8 to 6.4. He et al. developed photoresponsive nanogels, which utilize light to reversibly change the cross-linking density of nanogel particles [40]. Poly(ethylene oxide) and poly[2-(2-methoxyethoxy)ethylmethacrylate-co-4-methyl-[7-(methacryloyl)oxyethyloxy]coumarin] (PEO-b-P(MEOMA-co-CMA)) were synthesized. The reversible photo-cross-linking reaction was provided by the photodimerization of coumarin side groups under irradiation at λ >310 nm and the photocleavage of cyclobutane bridges under irradiation at λ <260 nm, reducing the degree of cross-linking and accelerating the rate of drug release.

**Table 1:** Main nanogel assembly techniques.

Assembly	Reactions	Properties	References
Physical	Micellar	Self-assembly using triblock copolymers or branched polymers	[31,34]
	Hybrid (nanoparticles suspended in hydrogels)	Nanoparticles immobilized in hydrogels	[35,41,42,43,44,45]
Cross-linking	Disulfide	Cross-linked via thiol-disulfide exchange reaction, Cleaved in response to glutathione	[36,37,46]
	Amide	High reactivity with carboxylic acids, activated esters, isocyanates and iodides	[38,47]
	Imine	Stable under physiological conditions and labile at acidic pH	[39,48]
	Photo-induced	Photo-induced cross-linking or cleavage	[40,49]

### 3.2. Desired Properties of Nanogels for Drug Delivery

An option to consider for the future treatment of brain tumors is a drug delivery system known as the nanogel. A nanogel is a three-dimensional hydrogel that is formed by connection of nano-scopic micelles dispersed within an aqueous medium (“nano-in-hydrogels”) having an inherent capability to incorporate hydrophobic molecules in the core of the micelles while maintaining a hydrophilic exterior [50]. Similar to hydrogels, nanogels are (i) mostly hydrophilic in nature, soft, resembling the texture of soft tissues, bioadhesive, biocompatible, and biodegradable. [51]. One of the most widely reported biocompatible nanogels are chitosan-based nanogels. Pereira et al. performed a thorough study of biocompatibility of a glycol chitosan nanogel, one of the highly biocompatible chitosan derivatives, in vitro [52]. Glycol chitosan nanogels did not induce noticeable metabolic activities and did not affect the cell membrane integrity in 3T3 fibroblast, HMEC human microvascular endothelial and RAW mouse leukemia monocyte macrophage cell lines. Glycol chitosan nanogels were poorly internalized by murine macrophages. Blood compatibility of glycol chitosan nanogels was confirmed by hemolysis and whole blood clotting time assays.

A number of nanogels demonstrate (ii) stimuli-responsive behaviors to release drugs in response to external stimuli [53]. One of the widely explored external stimuli is temperature. Some nanogels are designed to make a sol-to-gel transition at 37 °C, body temperature. Below 37 °C, nanogels are in a sol form. At 37 °C, nanogels begin to increase the viscosity, forming a semisolid gel. At ambient temperature, the viscosity of the sol is low allowing the formulation to pass through the syringe/needle. When injected, at 37 °C, nanogels are formed conforming to a shape of body cavity. Poly(ethylene oxide)-block-poly(propylene oxide)-block-poly(ethylene oxide) (PEO-b-PPO-b-PEO), also known as poloxamer, Pluronics, or Kolliphor, has been explored widely to create thermos-responsive nanogels [50,51,54]. Poloxamer 407 is a triblock copolymer with a hydrophobic central PPO core and two hydrophilic PEO side chains. At the concentrations of PEO-b-PPO-b-PEO of 20–30% *w*/*w*, the copolymers reach the critical micelle concentration; it is at this point where the micelles reorder themselves into a lattice [54]. Upon the elevation of the environmental temperature (at 37 °C), the hydrophilic chains are desolvated as the hydrogen bonds between the solvent and chains begin to break leading to chain entanglement. The resultant product is a gel that allows for the gradual release of hydrophobic drugs and the more rapid release of hydrophilic drugs in the insertion site. Thermo-responsive behavior of nanogels can also serve an ideal dermal drug delivery system. Gerecke et al. reported that thermo-responsive nanogels synthesized from dendritic polyglycerol with poly(glycidyl methyl ether-co-ethyl glycidyl ether) were capable of enhancing penetration through biological barriers such as the stratum corneum and were taken up by keratinocytes of human skin without cytotoxic or genotoxic effect [55]. 

Another desirable behavior of nanogels is pH sensitivity. Nanogels are designed to undergo the cleavage of the polymer networks/linkages under acidic conditions (mimicking tumor environment) and degrade completely, utilizing various cross-linkers, but are stable in physiological environment. Yang et al. developed a pH-triggered hyaluronic acid nanogel system by copolymerization between methacrylate hyaluronic acid and a cross linker containing ortho ester groups [56]. This nanogel system carrying doxorubicin demonstrated excellent tumor homing and selective tumor cell uptake, resulting in superior anticancer efficacy in HepG2 human liver cancer cell spheroids. The rapid release of doxorubicin was observed under endo/lysosomal conditions due to the pH-triggered cleavage of ortho ester linkages. Kang et al. developed a pH-responsive, chemically cross-linked nanogel using dopamine hydrochloride-conjugated carbonized hyaluronic acid [57]. Release of doxorubicin from this nanogel system was pH-dependent, resulting in 80% of doxorubicin released in 30 h at pH 5.0. Less than 20% of doxorubicin was released from nanogels at pH 6 and 7.4. This pH-dependency was caused by the cleavage of boronate ester bond between catechol and boronic acid under acidic conditions.

Multi-stimuli responsive nanogels have been shown to exhibit greater “fine-tuning” effect compared to their singular-stimuli responsive counterparts. Salehi et al. dual-stimuli responsive nanogels were composed of poly(N-isopropylacrylamide-dimethylaminoethyl methacrylatequaternary ammonium alkyl halide-methacrylic acid) and poly(N-isopropylacrylamide-dimethylami-noethyl methacrylate quaternary ammonium alkyl halide-meth-acrylic acid-hydroxyethyl methacrylate) [58]. This nanogel system showcased the capability of stimuli-triggered-controlled release behaviors mediated by temperature and pH values and were administered for the simultaneous delivery of doxorubicin and methotrexate. The release of both drugs were accelerated at pH 4 and 5.5 but arrested at pH 7.4. The release of both drugs was more rapid at 40 °C than at 37 °C. The authors highlighted that the prolonged and constant drug release pattern along with the dual-stimuli responsive behaviors offer a cancer treatment option without the frequent administration of multi-drugs. Pan et al. developed multi-stimuli responsive nanogels using the tailored modified sugarcane bagasse cellulose [59]. In the presence of a disulfide crosslinking agent, cystamine bisacrylamide, the in situ free radical copolymerization of methacrylated monocarboxylic sugarcane bagasse cellulose and N-isopropylacrylamide was processed, thus leading to redox (in the presence of reducing agent), pH (below 5.8) and temperature (above 32 °C)-responsive nanogels.

Nanogels are one of the excellent drug delivery systems with (iii) the capability of incorporating and delivering a wide range of drugs by immobilizing them through covalent or non-covalent interactions. Notably, nanogels demonstrate (iv) controlled the release of multi-drugs with the primary release mechanism being diffusion of drug followed by the degradation of polymeric matrix. Cho et al. investigated theragnostic effects of thermosensitive PLGA-b-PEG-b-PLGA nanogels carrying paclitaxel, rapamycin, an NIR imaging agent in ovarian cancer-bearing mice [60]. Nanogels made a sol-to-gel transition at 37 °C and slowly released drugs at a simultaneous release rate in response to the physical degradation of nanogels. Nanogels released c.a. 26% of the payloads within 48 h whereas a micellar liquid formulation released the payloads at more rapid rate, reaching 68–70% content release within 48 h. This thermos-responsive nanogel system enabled loco-regional delivery of multi-payloads by forming a gel-depot in the peritoneal cavity of ovarian cancer-bearing mice. In the control, without treatment, animals bearing ES-2- human ovarian cancer increased tumor burden significantly from 100% to 3480 ± 445% within 3 days. A single intraperitoneal (IP) injection of nanogels remarkably decreased tumor burden from 100% down to 7 ± 1% on day 3. A single intravenous (IV) or IP injection of micelles containing the same drugs did not show the therapeutic effectiveness, demonstrating increase of tumor burden from 100% to 110 ± 21% for IP micelles and 100% to 471 ± 236% for IV micelles. Cho et al. also developed 3D printed nanogel discs constructed of PEO-PPO-PEO and therapeutic payloads, paclitaxel and rapamycin [54]. The authors emphasized the convenience in use, proposing that in clinical settings, healthcare providers could place the disc into the peritoneal cavity post-surgical ovarian tumor removal without concerns of unsuccessful delivery of medications nor detrimental effects on patient recovery known as peritoneal adhesion.

Modified nanogels can (v) target the specific receptors or tissues. Su et al. synthesized thermo- and pH-responsive poly (N-isopropyl acrylamide-co-acrylic acid) nanogels [61]. Fluorescent bovine serum albumin (BSA) encapsulated gold nanoclusters were conjugated onto the surface of nanogels, followed by functionalization of tumor targeting peptide iRGD onto the BSA for tumor targeting. Nanoparticles carrying doxorubicin were c.a. 182 nm in diameter. The targeted nanogel system enhanced intracellular uptake of the payload, doxorubicin, into the vein endothelial (HUVECs) and the extravascular tumor (B16) cells. Chen et al. designed and constructed a smart nanogel platform integrating both receptor-mediated targeting (RMT) and environment-mediated targeting (EMT) strategies to heighten the tumor accumulation and cellular uptake of drugs [62]. A phenylboronic acid (PBA) and morpholine (MP) dual-modified polypeptide nanogel (PMNG) were prepared. The PBA ligand selectively targeted sialyl (SA) overly expressed on the highly metastatic tumor cells. The MP ligand favored extracellular pH condition (c.a. pH 6.5) and facilitated the internalization of drugs into cells. In vivo, this smart, targeted nanogel system carrying doxorubicin exhibited the outstanding efficacy of the inhibition of metastatic nodules in C57/BL mice bearing subcutaneous melanomas.

The aforementioned properties of nanogels make them outstanding candidates for pharmaceutical/biomedical applications as a drug delivery system, specifically involving brain cancer (Table 2).

## 4. Nanogels That Deliver Drugs to Brain

### 4.1. Nanogels That Cross the BBB

The ability to transport of compounds across the BBB is a fundamental requirement to treat and diagnose various brain diseases [63]. The BBB prevents compounds from reaching therapeutic concentrations in the brain, thereby hampering the therapeutic/diagnostic efficacy. Many studies have elucidated a few factors for compounds, especially nanoparticles, to penetrate the BBB and reach the brain [64]. Nanoscopic drug delivery systems can cross the BBB in a variety of ways, including endocytosis, receptor-mediated transcytosis, or the enhanced permeability and retention (EPR) effect [65]. The EPR effect exploits the leaky vasculature of solid tumors where the nanoparticles extravasate locally into the tumor tissues, resulting in slow release of encapsulated drugs into the brain tumor tissue. Intravenous injection of nanogels could be a potential method for drug delivery for brain tumors relying on the EPR effect.

The molecular and particle “sizes” and “hydrophilicity” of the compounds are considered to be predominantly important factors enabling their migration across these barriers [66,67,68,69]. Small, hydrophilic molecules may cross the BBB via paracellular transportation, but it may be limited by the regulation of the transient relaxation of tight junctions between the endothelial cells [70]. Small, lipophilic molecules enter the brain tissues via transcellular diffusion [70]. However, the route of transcellular diffusion involves the traversing of the luminal membrane and cytosol prior to reaching the brain tissue, which represents a challenge due to the tendency of lipophilic substances to remain within the cell membrane. Kimura et al. prepared ultra-fine hydrophilic nanogels (average particle size between c.a. 5 and 21 nm) carrying Gd-DOTA for brain imaging. In mice, it was confirmed that intravenously injected nanogels helped Gd-DOTA enter brain parenchyma through the BBB.

Ribovski et al. added one more advantage of nanogels in delivering compounds across the BBB, which is “low stiffness” [63]. The authors investigated the effect of nanogel stiffness on nanogel transport across the in vitro BBB model and calculated the fraction of internalized nanogels that reached the basolateral compartment of the BBB model. The softer nanogels showed a 2-fold higher secretion at the basal side of the BBB model compared to the stiff nanogels. The authors hypothesized that low nanogel stiffness promoted intracellular trafficking and transcytosis.

She et al. demonstrated that the “biocompatibility” of nanogels that mimic the cellular membrane was the key factor for effective drug delivery across the BBB [67]. The authors synthesized an azobenzene-based cross-linker to construct hypoxia-degradable zwitterionic phosphorylcholine nanogels. This nanogel was degradable in hypoxic environment, leading to the collapse of nanogels and rapid release of drugs in hypoxic tissue of glioblastoma. Nanogels were able to pass through the BBB and exhibited the high accumulation of the payload in glioblastoma tissue due to the phosphorylcholine mimicking cellular membrane. Nanogels were able to deliver doxorubicin effectively to the brain of mice and demonstrated the superior therapeutic behaviors in treating glioblastoma.

### 4.2. Nanogel Use in Brain Cancers

**Figure 2:**
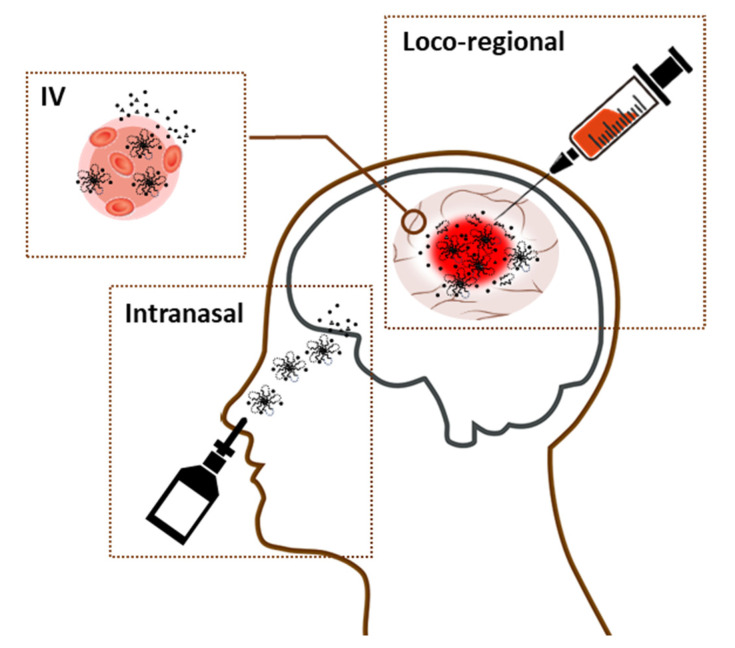
Administrations of nanogels that target brain.

Nanogels could be used during surgery; the removal of tumor tissue could be followed by the insertion of a nanogel, which would then harden and provide a protective layer or a filler of the resection cavity where the tumor was removed. In addition, nanogels could be loaded with multi-therapeutic agents in order to keep the tumor at bay for the foreseeable future (Figure 2). Unlike the wafers, the dose of loaded drugs can be easily controlled in a syringe based on the size/shape of resection cavity created by removing tumor tissues (Table 2). For nanogels, cutting or overlapping multiple units is not required. In addition to the loco-regional therapeutic benefits, some reports highlighted that diluted nanogels administered intravenously improved selective accumulation of nanogels in brain tumor tissues in vivo. Nanogels could also deliver therapeutic agents intravenously or intranasally to target brain tumor tissues prior to or after a surgical procedure.

Lin et al. prepared MRI traceable, rapidly gelating hydrogels by blending negatively charged carboxymethyl cellulose-grafted poly(N-isopropylacrylamide-co-methacrylic acid) and positively charged gadopentetic acid/branched polyethylenimine [71]. Hydrogels carried hydrophilic epirubicin and hydrophobic paclitaxel (PTX) incorporated in bovine serum albumin nanoparticles (BSA/PTX NPs: average diamer of 181.7 ± 3.9 nm). Hydrogels exhibited free-flowing sol phase at 4°C and made a transition to non-flowing gel phase at 37 °C. In vivo hydrogels carrying epirubicin and BSA/PTX NPs were implanted to the residual tumor tissues after surgical tumor resection in humam glioma U87 tumor-bearing mice. Hydrogels carrying epirubicin and BSA/PTX NPs showed remarkable tumor growth inhibition with the medium survival of 69 days. Notably, the average survival spans for animals in control group (receiving no treatment after surgical tumor removal) was c.a. 27 days. The authors presumed that nanogels facilitated “tumor-priming effect” by releasing epirubicin rapidly at the first stage to prime tumor tissues to maximue therapeutic efficacy of paclitaxel released later.

McCrorie et al. designed a spray delivery system consisting of a bioadhesive hydrogel (pectin) and poly(ethylene glycol)-block-polylactic acid (PEG-b-PLA) micelles carrying etoposide and olaparib (the average diameter of 70 nm) [72]. The release was rapid with a burst release of 5% for olaparib in the first 30 min followed by 85% after 24 h. For etoposide, there was a similar initial release of 9% in the first 30 min followed by 83% after 24 h. For both drugs, 100% of the drug was released after 48 h. A pectin-based hydrogel showed the potential to adhere to brain tissues due to the bioadhesive forces, instead of being washed away by the interstitial fluid. Following insertion of nanogels, pectin degrades slowly over 14 days within the brain. There was no neurotoxicity observed in mice. The authors also simulated surgical brain tumor removal followed by spray-delivering nanogels. While under general anesthesia, a small incision was made through the skin along the midline of the skull [72]. A larger drill bit was used to enlarge the burr hole to approximately 1–2 mm. Some brain tissues were removed and nanogels carrying drugs were sprayed into the linings of a surgical pseudo-resection cavity. Sequential biopsies were taken from below this cavity to determine successful delivery of the system and assess depth of penetration. The burr hole was then plugged with bone wax and the skin sutured shut. The authors observed the presence of nanoparticles in the surround tissues up to 1.5 cm away from the cavity. It was evident that sprayable hydrogels containing nanoparticles could be a great loco-regional treatment modality post-surgical brain tumor resection.

Picone et al. developed poly(N-vinyl pyrrolidone)-co-acrylic acid nanogels conjugated with insulin for intranasal delivery of insulin [73]. The average particle size of nanogels was c.a. 70 nm. Free insulin or the nanogel system carrying insulin was administered intranasally in mice, and the localization of insulin in the different parts of brain were analyzed. Nanogels increased the levels of insulin in the olfactory bulb, hippocampus and cerebral cortex at statistically greater levels at 30 and 60 min from intranasal administration. These results imply that the mucoadhesive properties of nanogels increased the retention time of insulin, facilitating muco-penetration of insulin. It also appears that insulin conjugated to nanogels was more resistant to the action of proteolytic enzymes in the nasal cavity. The literature has suggested that via intranasal application, drugs (low and large molecular weight drugs) can be transported into the brain via the olfactory and the trigeminal nerve pathways [73]. Drugs transported via the olfactory pathway enter the rostral area of the brain, whereas the trigeminal nerve route facilitates drug delivery to the caudal area of the brain. Although this nanogel system was not specifically used to treat brain cancer, it clearly presented that mucoadhesive nanogels were capable of augmenting the level of drugs in the olfactory bulb, hippocampus and cerebral cortex, benefiting the effective delivery of therapeutic agents across the BBB.

Shatsberg et al. designed disulfide crosslinked nanogels based on the polyglycerol-scaffold to deliver microRNA for glioblastoma therapy [74]. The primary amine groups with higher pKa in this nanogel system were protonated at neutral pH, imparting the positive charge to bind microRNA and facilitating cellular uptake via endocytosis. The secondary amines with lower pKa were protonated at endosomal pH, providing this nanogel system with endosomal escape capacities (via proton sponge effect). This nanogel system was designed to be cleaved under the intracellular reductive conditions. The polyplex formed between nanogels and microRNA were c.a., 140–170 nm in hydrodynamic diameter. Nanogels enabled internalization of microRNA into U-87 MG glioblastoma multiforme cells whereas intracellular localization of naked microRNA was hardly observed. U-87 MG glioblastoma multiforme-bearing mice received nanogels carrying microRNA intratumorally on days 0, 3, 7, and 10. Nanogels carrying microRNA helped restore the tumor suppressor role of miR-34a in the xenograft mice, resulting in remarkable inhibition of tumor growth. The authors stated that nanogels were chosen as microRNA delivery carrier for glioblastoma therapy due to their controllable size, shape, functionality, good mechanical properties, presence of the voids allowing encapsulation of multi-drugs, and tunability of drug release profiles resulting extended drug circulation time in the blood.

Azadi et al. developed nanogels using chitosan and polyanionic pentasodium triphosphate [75]. The nanogels carrying methotrexate was c.a. 118.54 ± 15.93 nm in diameter. The plasma and brain concentrations of methotrexate at different time points following intravenous administration of the diluted nanogels versus free drug provided the evidence that nanogels improved the efficacy of drug delivery in the brain. A 2.4-fold increase in drug plasma concentration and a 10–15-fold increase of drug concentration in the brain were obtained in male Sprague–Dawley rats as a result of the intravenous administration of methotrexate-loaded nanogels. The authors called this the “Trojan Horse” effect. The authors explained that this effect was presented due to the longer retention time of nanogels in the brain which, in turn, compensate for the drug efflux from the brain to the circulation.

Jiang et al. developed pH/temperature-sensitive poly(N-isopropylacrylamide-co-acrylic acid) nanogels carrying citric acid-coated Fe_3_O_4_ nanoparticles [76]. After conjugated with Cy 5.5-labeled lactoferrin, the resultant nanogels serve as bifunctional contrast agent for both MRI and intraoperative optical imaging for glioma. The nanogels had a mean hydrodynamic diameter of 95.5 ± 6.2 nm. In vivo application of IV nanogels on glioma detection with MRI and fluorescence imaging were evaluated in rats bearing C6 glioma. Nanogels appeared to selectively accumulate in the tumor tissues and could be used for the pre-operative MRI diagnosis of the glioma. The optical imaging ability of the nanogels was verified by acquiring ex vivo fluorescence images. A significant fluorescence signal was observed only in the brain tumor region of the rat receiving nanogels. The nanogels were proven to be biocompatible with no noticeable toxic effects detected in important biological functions and major organs.

## 5. Challenges and Prospects for Nanogel-Based Drug Delivery to Brain

Despite their possible novel uses, it is also important to address some setbacks of nanogel use. As aforementioned, a great property of nanogels is their ability to release drugs stimulated by external stimuli (e.g., temperature, pH, enzyme), but this property also has a downside. Should the nanogel arrive an environment where it can degrade to release drugs before it reaches the target site of action, significant problems can arise with delivery of a drug to the off-target, leading to adverse reactions. Considering the unque feature of nanogels releasing drugs slowly and gradually and their prolonged residence time in the body, the off-terget effect may exacerbate the adverse reaction.

Other limitations to nanogels include the particle size and polydispersity of nanocarriers and issues associated with polymer degradation. The impermeable characteristics of the BBB have been considered to be the main reason for the failure to achieve therapeutic drug concentrations in the brain tissue [77]. Especially, the BBB prevents many large molecules, including peptides and medicinal macromolecules, from entering the brain and the rest of the central nervous system. It is primarily because brain capillary endothelial cells are closely connected to each other by tight intercellular junctions and zonulae occludentes. Dehghankhold et al. indicated that the particle sizes of long-circulating drug delivery systems should range between 50 and 200 nm to deposit the systems in the brain [77]. Successful nanogel formulation requires the preparation of homogenous (polydispersity index PDI <0.7) nanocarriers of the average size of 50–200 nm, noting that very small particles (<0 nm) are rapidly cleared via the renal system. Nanogels are commonly formulated with synthetic polymers and organic/inorganic solvents. It is especially crucial to investigate the toxicity of the degraded polymers in the brain and make sure to remove toxic solvent completely from the formulation. It is important to modify the polymers to maximize their bioadhesiveness, biocompatibility and biodegradabiliy. It is also ideal to design the nanogels wisely to minimize any toxic effects caused by fragmented/cleaved polymers after degradation of nanogels.

One of the last major challenges is the inconvenience related to handling and storage. This applies specifically to temperature-responsive nanogels with the gelation temperature lower than 37 °C. This may cause several issues including the premature gelation in the syringe/needle, instability problems affecting the product shelf-life and difficulty in handling.

Considering experimental results from the published articles and experts’ opinions/reviews, we listed a few key desirable properties of nanogels carrying drugs that can help seamlessly incoporation of nanogel systems in brain cancer treatment regimen (Table 3). These include the tissue-like properties of gels, particle sizes of 50–200 nm and polydispersity index of nanoparticles below 0.7, the capability of loading multiple agents and releasing agents when desired at controlled and gradual manners, desired rheological patterns, prolonged retention of nanogels in the patient’s body, and the storage stability.

## 6. Conclusions

Nanogel technology presents an opportunity for viable, lucrative, and efficient future treatments of brain cancer. Brain cancer treatment’s critical obstacles are the BBB, diversity of intracranial neoplasms, and the complexity of the organ in which it resides, limiting the treatment options. Nanogels provide local or systemic treatment options that respect the BBB and the physiological feature of the cranial cavity while limiting adverse effects.

## Figures and Tables

**Table 2:** Comparison of nanogels, wafers, and liquid dosage forms for their properties as drug delivery systems for brain cancer.

Properties	Nanogels	Wafers	Nanoparticle-Based Liquid Dosage Forms
Route of administration	IV, implant, intratumoral, nasal	Implant	IV, intratumoral, nasal
Multi-drug delivery	Yes	Maybe (not known)	Yes
Delivery of hydrophobic drugs	Yes	Maybe (not known)	Yes
Form of drugs	Encapsulated in nanoparticles	Free form	Encapsulated in nanoparticles
Dose adjustment	Yes (via syringe)	Yes but manipulation needed (e.g., cutting, inserting multiple wafers)	Yes (via syringe)
Surface modification for targeted drug delivery	Yes	No	Yes
Long residence time	Yes	Yes	No
Controllable drug release	Yes (stimuli-responsive, diffusion followed by physical degradation)	Yes (physical erosion)	Yes (stimuli-responsive, diffusion followed by physical degradation)
Suitable for intratumoral injection	Yes	No	Yes
Available as a spray delivery system	Yes	No	Yes
Biocompatible and biodegradable	Yes	Yes	Yes
Convenience in handling	+	+++	++
Conforming to the shape/size of the resection cavity post-surgery	Yes (intimate contacting with surrounding tissues)	No (stiff)	No (easily washed away by the interstitial fluid)

**Table 3:** List of key desirable properties of nanogels desired for brain cancer therapy.

Properties	Nanogels
Gels	Bioadhesive, biocompatible, biodegradable, soft “tissue-like” texture, able to conform to the shape/size of the resection cavity
Nanoparticle size	50–200 nm with PDI <0.7
Payloads	Multiple (hydrophilic and hydrophobic) agents (therapeutics and/or diagnostics)
Drug release	Demonstrate controlled and gradual release of drugs only when exposed to stimuli (e.g., pH, enzyme) Demonstrate simultaneous or sequential release of multi-drugs Minimize premature drug release
Rheology	Maintain the viscosity under shear stress and at storage/handling Design the system to increase the viscosity only when exposed to specific stimuli (e.g., temperature)
Modification	Conjugate targeting moiety and/or imaging agents Include polymers that maximize biodegradability and biocompatibility
Gelation	Make a sol-to-gel transition rapidly by responding to stimuli
Retention time	Retain extended period of time to increase drug concentrations in plasma and brain tissues
Degradation	Degrade rapidly when no longer needed Leave no residual polymers Does not produce toxic byproduct/degraded polymer fragments
Administration	Exhibit the versatility in routes of administration (e.g., loco-regional, intravenous, and intranasal)
Storage	Maintain product stability at storage Does not require special storage conditions (e.g., freezer)
